# Odontalgia

**Published:** 1838

**Authors:** S. P. Hullihen

**Affiliations:** Wheeling, Va.


### Art. VI.—Odontalgia.

*Observations on Toothache.* By S. P. Hullihen, of Wheeling, Va.

There is no disease, perhaps, which attacks the human family so indiscriminately and universally, as the toothache; none which the physician is called upon so often to alleviate, and none that he treats with less success.

There can be few things in medicine more erroneous, than to call every painful affection of the teeth "toothache," and treat them all as one specific disease. As well might a wound in the scalp, a fracture of the skull, or inflammation of the brain, be called "headache," and treated as such.

Toothache most generally originates from local causes, differing sometimes materially from each other. However, pains are ofttimes experienced in the teeth, through that mysterious agent usually termed sympathy, when the exciting cause may exist in a distant part of the system. Females are particularly liable to be affected in this way, during pregnancy.

A painful tooth is always annoying, and sometimes exceedingly severe; but this is trifling, compared with the consequences that frequently ensue from it. Diseases of the most malignant nature, and deformities the most unseemly to be beheld, are not unfrequently the result of toothache, or rather, of the disease which occasions it.

With these remarks, I shall proceed to treat, separately, of the different causes of toothache, in the following order:—

1st. Toothache from exposure of the nerve;

2d. From fungus of the nerve;

3d. From the formation of pus in the internal cavity of a tooth;

4th. From a diseased state of the external membrane covering the fang;

5th. From sympathy.

TOOTHACHE FROM EXPOSURE OF THE NERVE.

The nerve of a tooth is intended by nature to be completely protected by a thick bony covering, and when exposed either by accident or disease, it becomes inflamed and painful, from the action of air, food, fluids, and other irritating causes.

The first attack of toothache from exposure of the nerve, is usually instantaneous, and is most generally brought on by some mechanical violence; either while eating, picking the tooth, preparing the cavity for a plug, from the fracture in attempting to extract a tooth, or otherwise. The pain is very severe, and rather of a sharp and darting character. It is easily palliated by any stimulating applications; but will immediately return, if air, food, or drinks of any kind, be suffered to come in contact with the nerve. The nerve will sometimes remain in this situation for weeks; but at length a high inflammation will ensue, and suppuration be the result, together with its total destruction of the tooth.

*Treatment.*—As this kind of toothache is always very troublesome, many remedies have been employed for the purpose of destroying the nerve, such as caustics, acids, and other applications of a similar nature, but without the desired effect. The actual cautery has also been resorted to, and has proved equally unsuccessful, owing to the utter impracticability in many cases, and the impossibility of applying so small an instrument with the necessary degree of heat. The essential oils, opium, creosote and various other stimulating remedies, have been used as palliatives, and with general success, when actually applied to an exposed nerve; but from the very prevalent opinion, that all pains of the teeth proceed from an exposure of the nerve, these remedies are often employed, when it is not in the nature of things, that they should prove in the least degree beneficial.

The most efficient method of destroying the nerve, and one, which from the nature of the operation, and the smallness of the instrument employed, can be performed with equal success upon any tooth in the mouth, is to rupture the blood-vessels of the nerve. This is done by taking a small wire, a little larger than a bristle, and plunging it into the nerve, and down the fang as far as possible. Upon withdrawing the instrument, a few drops of blood will follow. The patient is quickly and permanently relieved from pain, and a speedy suppuration of the nerve is always induced.

There is another method, which I have found very successful; but it is objectionable from the great length of time it frequently requires to effect a cure. This consists in the wearing of lint in the cavity of the tooth, wetted with creosote, or some of the essential oils, until the nerve suppurates. The application is to be renewed every day, taking care that the lint does not produce the least pressure upon the nerve. This treatment usually prevents the tooth from aching, and likewise promotes the suppuration of the nerve, and suppurate, every exposed nerve must. It is an operation Nature sets up for its removal.

Whenever there is a discharge of pus through the crown of a tooth, from the internal cavity, care should be taken to prevent the opening from being closed with food; for when this takes place, the tooth becomes very sore and painful. Therefore, lint wetted as before directed, should always be kept in the cavity; it will absorb the matter, and likewise prevent such an occurrence.

### TOOTHACHE FROM FUNGUS OF THE NERVE.

Fungus of the nerve, so called from its occupying the former situation of the nerve, most probably originates from a remnant of the dental blood-vessels. It invariably attacks fangs and teeth, where the nerve has been destroyed for a long period; it is of a deep red color; very soft; bleeds freely upon the slightest touch; is sometimes totally insensible; at others highly sensitive; giving much pain of an incessant character; entirely free from any darting or throbbing sensation; and when small and painful, is frequently mistaken for an exposed nerve. It varies in size, from that of a pin's head to a pea, or larger. It is sometimes, when small, deeply situated in a fang; but more generally protrudes, filling up a cavity formed by caries. It always occasions a most fœtid and disagreeable breath, which no spice nor perfume can conceal, nor art remove, as long as it is suffered to remain.

*Treatment.*—To destroy this diseased growth is very difficult, and I may say impossible, except in such cases which permit the actual cautery to be applied to its root; and, as the disease commences in the very foramen of the internal cavity of the fang, this means is narrowed down to a few cases, when at all practicable. When a tooth becomes painful from this affection, it is only necessary to make it bleed freely, to ensure immediate relief; but in all cases where the fungus cannot be completely eradicated, the tooth should be extracted, for there is no disease of the mouth which makes the breath so intolerable. Some time since, a young lady, about 17 years of age, applied to me with a growth of this kind, in each of the second molares of the under jaw, which had assumed rather a novel character. She stated that the fungi had made their appearance in both teeth at the same time, about four years before, and that for the last two years she had been much troubled with a bleeding from them, which took place regularly once a month, and continued from two to four days. She being very anxious to have the teeth saved, I destroyed, to all appearance, the morbid growth, and plugged the teeth. In a few days, they became sore and painful; the plugs were removed, and a slight bleeding commenced, which continued for three days, and then the tumors disappeared entirely. I was therefore induced to plug them again; but, in about three weeks, the teeth became sore, the plugs were removed, and a bleeding ensued as before. I now suspected it to be a *vicarious menstruation*, and mentioned the case to the family physician. At his request, I plugged them again, and the result was precisely as before. The teeth were then removed, and the patient was put under a course of treatment, by her physician, which effected a cure.

### TOOTHACHE FROM THE FORMATION OF PUS IN THE INTERNAL CAVITY OF A TOOTH.

Whenever the nerve of a tooth is rendered near the external surface by caries, fractures, or from the absorption and receding of the alveolar process and gum, or other causes, it is subject to much irritation, varying however in proportion to the extent and loss of covering. Thus a greater or lesser inflammation of the nerve necessarily ensues, producing the following very different effects. When the inflammation is slight, and comes on gradually, some dormant vessels are stimulated into action. A bony deposite takes place in the internal cavity of a tooth, and the nerve is again completely protected from all external irritation. This more frequently occurs in teeth that are worn down by constant friction. The entire crowns of teeth are sometimes worn off, and the nerve still protected by this beautiful process of nature. But in the great majority of cases, inflammation is induced rather speedily, and runs sooner or later into excess. Suppuration of the nerve is the consequence, and from this result may be traced the true causes of many diseases of the jaws and mouth not generally understood.

Toothache from suppuration of the nerve occurs very frequently. At first, the tooth is painful, only when hot or cold fluids or food pass over it, and may continue in this situation for some time. At length a steady gnawing pain supervenes, and then the tooth begins to get sore, and appears a little loose and longer than the rest. About this period, the pains assume a very different character, darting from the tooth along the courses of the nerves, to the temple, ear and side of the head, and to the teeth of both jaws on that side. Within twenty-four hours, the face begins to swell, and the pain changes to one of a constant throbbing description, and appears to beat in unison with the pulse; symptoms which indicate that an alveolar abscess is forming.

When a tooth, in the manner described, becomes sore, the nerve is about suppurating; when it appears larger than the rest, loose, and is very painful, the nerve has suppurated, and the pus is beginning to ooze out at the extremity of the fang, where the vessels enter, producing much pressure, hence the sharp darting paroxysms. When the cheek begins to swell, the matter is effusing between the alveolus and the lining membrane, and occasions the throbbing pain which always accompanies the formation of an alveolar abscess. The true abscess of the antrum maxillare originates, in like manner, from the same cause, when the fangs are very near, or penetrate that cavity.

There is now and then a case, that differs materially from the general progress of this disease. The nerve may suppurate, and the face swell, and both pain and swelling subside, without the development of an abscess. When this occurs, the matter is insinuated between the end of the fang and the membrane covering it, about the size of a pea, and sometimes larger, which may be felt distinctly in the gum, opposite the end of the fang. By pressing on it, a fulness, or sensation of pressure, is felt in the tooth. But this sac will eventually burst, and an abscess is invariably the result.

*Treatment.*—As soon as it is ascertained, that the nerve is suppurating, the tooth should be *trepaned.* The matter is thereby suffered to escape as soon as formed, and the patient is immediately relieved from pain. In such cases, when the matter has even begun to ooze out at the end of the fang, and the face has not yet become swollen, the same treatment should be observed, and will most generally prove successful. But when the face is already swollen, and the pain is of a throbbing character, there is no remedy but the extraction of the tooth.

The proper place to select for trepaning or drilling a tooth, depends altogether on the cause that produced the suppuration of the nerve. If it be from caries, a hole should be drilled from that cavity; if from a fracture, or the wearing down of a tooth, the nearest point to the nerve should be selected; if from too great exposure of the fang, from absorption of the alveoli and gums, and the tooth be sound, it should be drilled from the inner side, as the matter may then be immediately sucked out, if necessary.

### TOOTHACHE FROM A DISEASED STATE OF THE MEMBRANE COVERING THE FANG.

Every practitioner will observe in the course of his practice, that at times toothache rages almost like an epidemic. This more frequently occurs when any very sudden change of weather takes place, particularly to cold and wet. The pain is mostly confined to fangs and teeth, in which the nerve has suppurated long before, causing alveolar abscesses; and when there has been a discharge of pus from the alveolar cell, occasioned by receiving a stroke on the tooth. In either case, an incurably diseased state of the membrane covering the fang is induced, and ever after it is liable to become inflamed and painful, on taking a sudden cold. Such fangs and teeth, almost every individual possesses, and being acted on at the same time, by the same cause, will account for the occasional *prevalence* of toothache. The pain is generally of a dull, heavy character, the tooth becomes a little sore, the gums very much inflamed, and of a bluish tinge immediately over and along the whole length of the fang. Sometimes the gum is puffy opposite the end of the fang, and the pain of a throbbing description. In such cases, matter is forming, and a partial filling up of an old abscess takes place, forming what is usually termed a "gum boil." It is rarely, if ever, that the face becomes swollen from an attack of this kind of toothache.

*Treatment.*—The pain may be greatly palliated, by making an incision through the gum, along the entire length of the fang, and then applying a roasted fig, or bruised raisins to the gum; but as the pain is almost sure to return upon taking cold, the extraction of the tooth should always constitute the treatment of this variety.

TOOTHACHE FROM SYMPATHY.

It is well known that the human system is sympathetically connected, in all its parts, by the nervous system; and yet some organs, from being more intimately connected than others, sympathize oftener than others.

During the formation of an alveolar abscess, there is much pain and inflammation in the offending tooth and surrounding parts, the nerves become involved and irritated, the irritation extends to all the teeth of both jaws on that side, and to the ear, temple and scalp. Sometimes each tooth sympathizes in its turn, so that some one is always more severely affected for a short time than the rest, and this more pungent sensation, is continually changing from one tooth to another, and to other associated parts.

Sympathetic toothache occurs very frequently during pregnancy, and is evidently induced, sometimes from an increased, at other times from a diminished action, dependent on the peculiar state of the general system. But in either case, the teeth are almost invariably diseased, and the painful sensation exclusively confined to them. Sufficient indications, however, are always present, and so distinctly marked, as to enable the practitioner at once to determine whether the pain proceeds from torpor or an inflammatory action.

*Treatment.*—When toothache is induced by direct sympathy with a diseased tooth, or the results arising from one, the cause, if possible, should be removed; but if the exciting cause exists in a distant part of the system, blood-letting and purgatives will be most generally indicated, and may be strongly recommended. But when a painful sensation is produced, from want of action or natural stimulus, the teeth *should not be extracted*, unless when so much decayed as to render them useless in mastication. For many useful teeth are thus affected, and are always easily relieved and oftentimes entirely cured, by any highly stimulating application to them and the gums; such as the essential oils, creosote, or a divided fig powdered over with pulverized camphor, and equal parts of cayenne pepper and mustard. But to brush the teeth and gums frequently every day, with a stiff brush dipped into some stimulating wash, is also very advantageous, and, perhaps, more generally efficient than any other remedy.

Exostosis is another disease of the teeth, which occasions the most intense and excruciating paroxysms; but as the pain is not generally felt in the tooth, nor its proximate parts, it cannot with propriety be considered under the head of toothache, and may therefore form the subject of some future paper.

Having now pointed out a number of causes producing toothache, all differing very materially from each other, and the treatment respectively required, I trust that the great impropriety of extracting every painful tooth, and *the utter impossibility of any remedy being discovered or invented, to cure all kinds of toothache*, will be clearly comprehended and duly appreciated.

January 1838.

